# Quantification of pulmonary perfusion abnormalities using DCE-MRI in COPD: comparison with quantitative CT and pulmonary function

**DOI:** 10.1007/s00330-021-08229-6

**Published:** 2021-09-22

**Authors:** Marilisa Schiwek, Simon M. F. Triphan, Jürgen Biederer, Oliver Weinheimer, Monika Eichinger, Claus F. Vogelmeier, Rudolf A. Jörres, Hans-Ulrich Kauczor, Claus P. Heußel, Philip Konietzke, Oyunbileg von Stackelberg, Frank Risse, Bertram J. Jobst, Mark O. Wielpütz, on behalf of the COSYCONET study group

**Affiliations:** 1 Department of Diagnostic and Interventional Radiology, Subdivision of Pulmonary Imaging, University Hospital of Heidelberg, Im Neuenheimer Feld 420, 69120 Heidelberg, Germany; 2 Boehringer Ingelheim Pharma GmbH & Co. KG, Birkendorfer Strasse 65, 88397 Biberach an der Riß, Germany; 3 Translational Lung Research Center Heidelberg (TLRC), German Lung Research Center (DZL), Im Neuenheimer Feld 156, 69120 Heidelberg, Germany; 4 Faculty of Medicine, University of Latvia, Raina bulvaris 19, Riga, 1586 Latvia; 5 Faculty of Medicine, Christian-Albrechts-Universität Zu Kiel, 24098 Kiel, Germany; 6 Department of Diagnostic and Interventional Radiology with Nuclear Medicine, Thoraxklinik at the University Hospital of Heidelberg, Röntgenstr. 1, 69126 Heidelberg, Germany; 7 Department of Medicine, Pulmonary and Critical Care Medicine, Philipps-University of Marburg (UMR), Marburg, Germany; 8 Institute and Outpatient Clinic for Occupational, Social and Environmental Medicine, University Hospital, Ludwig Maximilians University (LMU) Munich, Comprehensive Pneumology Center Munich (CPC-M), Munich, Germany

**Keywords:** Perfusion imaging, Biomarkers, Magnetic resonance imaging, Chronic obstructive pulmonary disease, Pulmonary emphysema

## Abstract

**Objectives:**

Pulmonary perfusion abnormalities are prevalent in patients with chronic obstructive pulmonary disease (COPD), are potentially reversible, and may be associated with emphysema development. Therefore, we aimed to evaluate the clinical meaningfulness of perfusion defects in percent (QDP) using DCE-MRI.

**Methods:**

We investigated a subset of baseline DCE-MRIs, paired inspiratory/expiratory CTs, and pulmonary function testing (PFT) of 83 subjects (age = 65.7 ± 9.0 years, patients-at-risk, and all GOLD groups) from one center of the “COSYCONET” COPD cohort. QDP was computed from DCE-MRI using an in-house developed quantification pipeline, including four different approaches: Otsu’s method, k-means clustering, texture analysis, and 80^th^ percentile threshold. QDP was compared with visual MRI perfusion scoring, CT parametric response mapping (PRM) indices of emphysema (PRM_Emph_) and functional small airway disease (PRM_fSAD_), and FEV1/FVC from PFT.

**Results:**

All QDP approaches showed high correlations with the MRI perfusion score (*r* = 0.67 to 0.72, *p* < 0.001), with the highest association based on Otsu’s method (*r* = 0.72, *p* < 0.001). QDP correlated significantly with all PRM indices (*p *< 0.001), with the strongest correlations with PRM_Emph_ (*r *= 0.70 to 0.75, *p* < 0.001). QDP was distinctly higher than PRM_Emph_ (mean difference = 35.85 to 40.40) and PRM_fSAD_ (mean difference = 15.12 to 19.68), but in close agreement when combining both PRM indices (mean difference = 1.47 to 6.03) for all QDP approaches. QDP correlated moderately with FEV1/FVC (*r* = − 0.54 to − 0.41, *p* < 0.001).

**Conclusion:**

QDP is associated with established markers of disease severity and the extent corresponds to the CT-derived combined extent of PRM_Emph_ and PRM_fSAD_. We propose to use QDP based on Otsu’s method for future clinical studies in COPD.

**Key Points:**

*• QDP quantified from DCE-MRI is associated with visual MRI perfusion score, CT PRM indices, and PFT.*

*• The extent of QDP from DCE-MRI corresponds to the combined extent of PRM*_*Emph*_ *and PRM*_*fSAD*_ *from CT.*

*• Assessing pulmonary perfusion abnormalities using DCE-MRI with QDP improved the correlations with CT PRM indices and PFT compared to the quantification of pulmonary blood flow and volume.*

**Supplementary Information:**

The online version contains supplementary material available at 10.1007/s00330-021-08229-6.

## Introduction

COPD is characterized by progressive airflow limitation caused by airway obstruction and emphysematous lung destruction. The resulting regional alveolar hypoxia leads to hypoxic pulmonary vasoconstriction (HPV), reducing pulmonary perfusion regionally. Moreover, the alveolar-capillary bed and pulmonary vessels are obliterated by emphysematous destruction. Both processes, HPV and the loss of lung capillaries, become apparent on functional imaging as regional perfusion abnormalities. DCE-MRI is an established technique to assess regional perfusion abnormalities by exploiting the contrast enhancement in lung parenchyma during the first pass of an i.v. injected contrast agent (CA) bolus. In clinical studies, perfusion abnormalities using DCE-MRI are assessed either by visual scoring systems or by computational methods for quantitative evaluation [1, 2]. The use of semi-quantitative scoring systems to monitor treatment effects on pulmonary perfusion might be challenging in early clinical studies designed to test treatments that slow emphysema progression. Due to the relatively slow disease progression in COPD and the short follow-up-intervals (up to 3 months) in early clinical studies, only relatively subtle changes in pulmonary perfusion are expected in such settings, which may remain undetected by scoring systems [3].

In previous studies, pulmonary blood flow (PBF) and pulmonary blood volume (PBV) were used to quantify pulmonary perfusion from DCE-MRI [1, 4, 5]. Particularly interesting for clinical studies, the quantification by computer algorithms limits human interaction and generates potentially more objective results than visual assessments. However, DCE-MRI in the lung is adversely affected by low contrast-to-noise ratios, non-linearities of the CA-signal relationship [6, 7], and pronounced image artefacts, which impair the reproducibility and robustness of PBF and PBV [8, 9]. The introduction of unsupervised image clustering algorithms on Fourier decomposition (FD), hyperpolarized helium, and xenon MRI enabled the quantification of the extent of perfusion or ventilation abnormalities relative to the lung volume (“defect-percent”) [10–13]. It has been shown that “defect-percent” is a sensitive marker for disease severity based on the observed correlations with spirometry, multiple breath washout, and morphological abnormalities [14–16]. However, different “defect-percent” quantification approaches were used and studies using DCE-MRI or their application in sizeable datasets are mostly missing.

The aims of this work were to (a) develop a robust algorithm to quantify QDP using DCE-MRI by comparing different approaches and (b) investigate the clinical meaningfulness of QDP by comparing it with a visual MRI perfusion score, quantitative CT PRM indices of emphysema, and functional small airways disease (fSAD), and PFT. Furthermore, we compared the performance of QDP with that of PBF and PBV.

## Materials and methods

### Study design and study population

The study is based on data from a prospective longitudinal multicenter imaging sub-study of the COPD cohort “Impact of Systemic Manifestations/Comorbidities on Clinical State, Prognosis, Utilisation of Health Care Resources in Patients with COPD”-trial (COSYCONET (NCT01245933); substudy “Image-Based Structural and Functional Phenotyping of the COSYCONET Cohort Using MRI and CT” (MR-COPD, NCT02629432)). The inclusion and exclusion criteria of COSYCONET can be found elsewhere [17]. In addition to subjects of the GOLD 1–4 categories [18], smokers and former smokers with no assignable GOLD category including the former GOLD 0 and subjects at risk for COPD were enrolled. The “former GOLD 0” group includes subjects with normal PFT in terms of FEV1/FVC (ratio between forced expiratory volume in 1 s and forced vital capacity) but with COPD-specific symptoms and the “at risk for COPD” group includes subjects not classifiable within GOLD, with normal PFT in terms of FEV1/FVC and without COPD-specific symptoms. Institutional ethics committee approval was obtained, and all subjects gave their written informed consent.

For this work, a subset of 103 subjects examined at the same center was selected and only baseline data were used.

### MRI acquisition

All subjects underwent MRI using the same 1.5 T scanner (Magnetom Aera, Siemens Healthineers) using a standardized chest MRI protocol (Supplementary table 1) [3, 19, 20], validated by a phantom study [21]. For the DCE-MRI perfusion imaging, a time-resolved T1-weighted 3D keyhole pulse sequence (time-resolved angiography with interleaved stochastic trajectories [TWIST]) with a fixed dose of 2 ml gadolinium-based CA (Gadobutrol, Bayer Vital GmbH) was injected i.v. at 4 ml/s followed by a saline chaser [22, 23]. The DCE-MRI (~ 33 s, temporal resolution ~ 1.6 s) was acquired in inspiratory breath-hold.

### MRI perfusion score

Visual scoring of DCE-MRI was performed by an experienced radiologist using a validated MRI perfusion score [2, 3, 20, 24]. The extent of perfusion abnormalities was assessed on lobe level as follows: 0 = no abnormality, 1 = < 50% of the lobe involved, and 2 = ≥ 50% of the lobe involved and summed for the whole lung, resulting in a maximum range between zero and twelve.

### MRI quantification pipeline

The in-house developed MRI analysis pipeline was written in MATLAB (R2019a, The MathWorks, Inc.). All image process steps were performed fully automatically unless otherwise specified. Further details are provided in the Supplementary materials.

#### Image pre-processing

Time-resolved subtraction images of the DCE-MRI were generated by subtracting the mean of the two first pre-contrast images. Arterial input functions (AIFs) were calculated in the pulmonary artery [25]. Time-resolved residue function maps (R(t) map) were computed by deconvolving voxel-by-voxel the AIF with each voxel of the subtraction image using truncated singular value decomposition [26]. The model used for the R(t) map calculation is based on the principles of tracer kinetics for non-diffusible tracers [27, 28]. Minor respiratory motion artefacts, usually occurring near the diaphragm, were excluded using cross-correlation analysis.

#### Quantification of pulmonary perfusion abnormalities

The lungs were automatically segmented from coronal T1-weighted images [29], registered to DCE-MRI images, and reviewed individually by an investigator. For comparison with the visual perfusion score, an approximate division of each lung into lobes was performed (Supplementary Fig. 1).

**Fig. 1:**
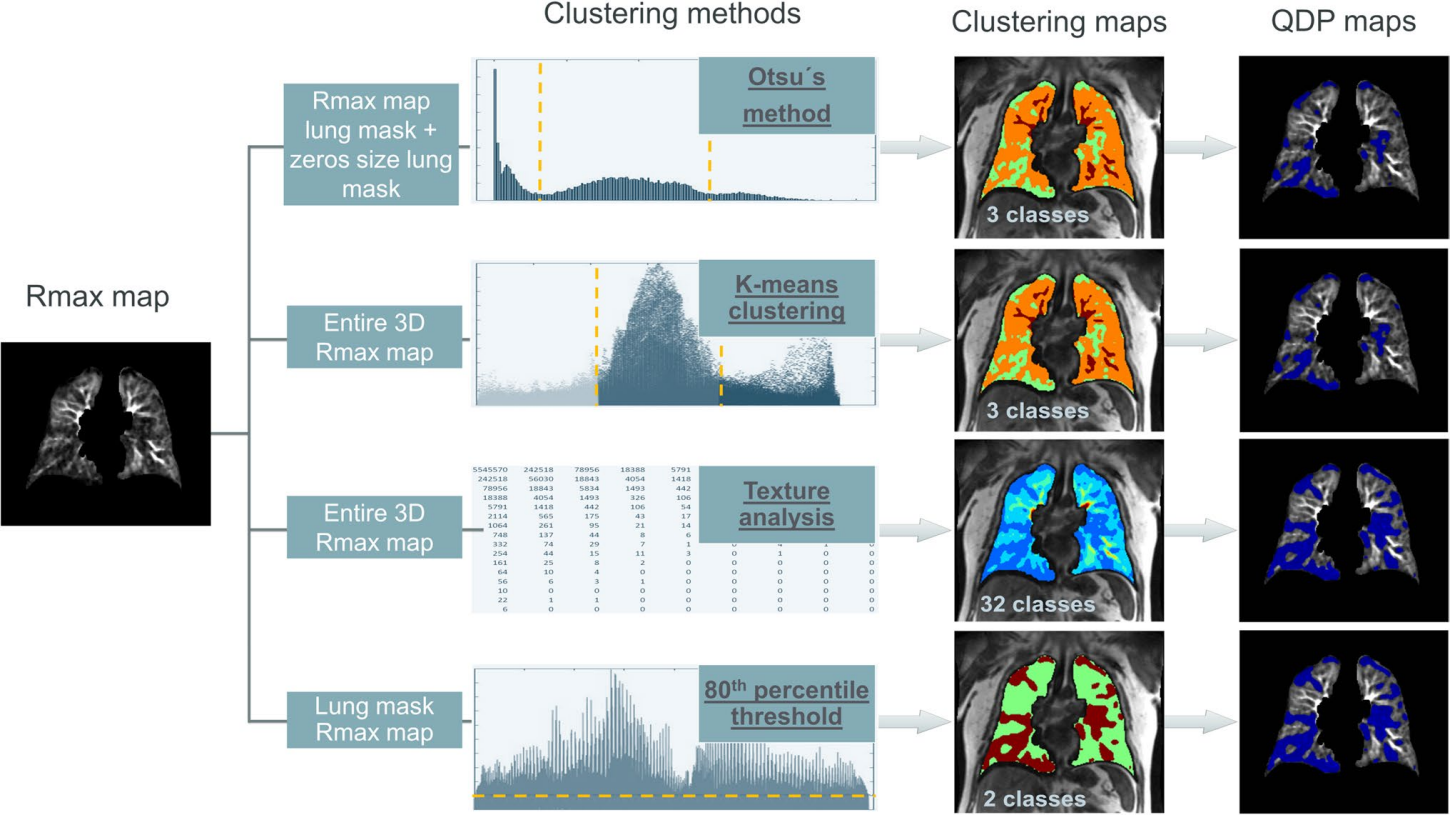
Flowchart describing the four different approaches to calculate perfusion defects in percent (QDP) using residue function map at the time point of maximum contrast enhancement (*R*_max_ map). Otsu’s method was used to find two thresholds from the histogram containing the intensity values of all voxels within the lung mask of the *R*_max_ map and zeros in the same amount as the lung mask's size, resulting in three classes (color map clustering: green = perfusion defects, orange = well perfused, red = vessels). K-means clustering was applied to the entire *R*_max_ map to separate the voxels’ intensities into three classes (color map clustering: green = perfusion defects, orange = well perfused, red = vessels). The texture analysis utilized the co-occurrence matrix of the entire *R*_max_ map to separate the voxels into 32 classes (color map clustering: each class with a different color from blue (perfusion defects) to red (vessels)). The 80^th^ percentile of all voxels' intensity inside the lung mask multiplied with 0.5 was used as a threshold between well- and poorly-perfused lung voxels, resulting in 2 classes (color map clustering: red = perfusion defects and green = well perfused). In all approaches, the class with the lowest mean signal intensity from the *R*_max_ map was defined as perfusion defect (= QDP). Corresponding QDP maps show resulting perfusion defects in dark blue. Further information about the different QDP calculation approaches can be found in the Supplementary materials

Pulmonary perfusion abnormalities in DCE-MRI are characterized by absent or delayed contrast enhancement. QDP was quantified from R(t) maps at the time point of maximum contrast enhancement (R_max_ map) using four different approaches based on unsupervised image clustering algorithms: Otsu’s method, k-means clustering, texture analysis and percentile-threshold. It was calculated in percent representing the extent of perfusion abnormalities relative to the segmented lung volume, with a theoretical range between 0 and 100%. Details about the four different calculation approaches are outlined in Fig. 1 and in the Supplementary materials.

For the comparison between QDP and the MRI perfusion score by lobe, QDP was converted into discrete values at lobe level (Supplementary table 2) [30]. The quantification of the pulmonary perfusion metrics PBF and PBV was based on the R(t) map as described elsewhere [4].

### CT parametric response mapping

All subjects underwent standardized phantom-controlled (Catphan600, The Phantom Laboratory) same-day non-enhanced low-dose CT (Somatom 64, Siemens Healthineers) with paired scans in inspiratory and expiratory breath-hold at 120 kV and 35 mAs. Images were reconstructed at 1.0 mm slice thickness with a 0.5-mm interval using a soft filtered back-projection convolution kernel (B30f). CT scans were post-processed using the in-house software YACTA as described previously (version 2.8.7) [31, 32]. PRM classified the lung in normal (PRM_Normal_), emphysematous (PRM_Emph_), or fSAD (PRM_fSAD_) [33]. PRM was calculated in percent relative to the segmented lung volume. PRM_Abnormal_ was computed to describe the proportion of non-normal lung tissue as PRM_Abnormal_ = PRM_Emph_ + PRM_fSAD_.

### Pulmonary function testing

PFT was performed according to the American Thoracic Society and European Respiratory Society recommendations [34]. In this study, forced expiratory volume in 1 s percent predicted (FEV1%predicted) and FEV1/FVC were used.

### Statistical analysis

Statistical analyses were performed using R (R 3.3.2, Foundation for Statistical Computing). Data are presented as mean ± standard deviation. Bland–Altman analysis, linear regression, Spearman correlation, Cohen’s k, percent agreement, Wilcoxon signed-rank test, Pearson and Filon’s z, one-way analysis of variance (ANOVA), and scatterplots were used. A *p* value < 0.05 was considered statistically significant. Further details are provided in the Supplementary materials.

## Results

### Patient population and technical feasibility

**Fig. 2:**
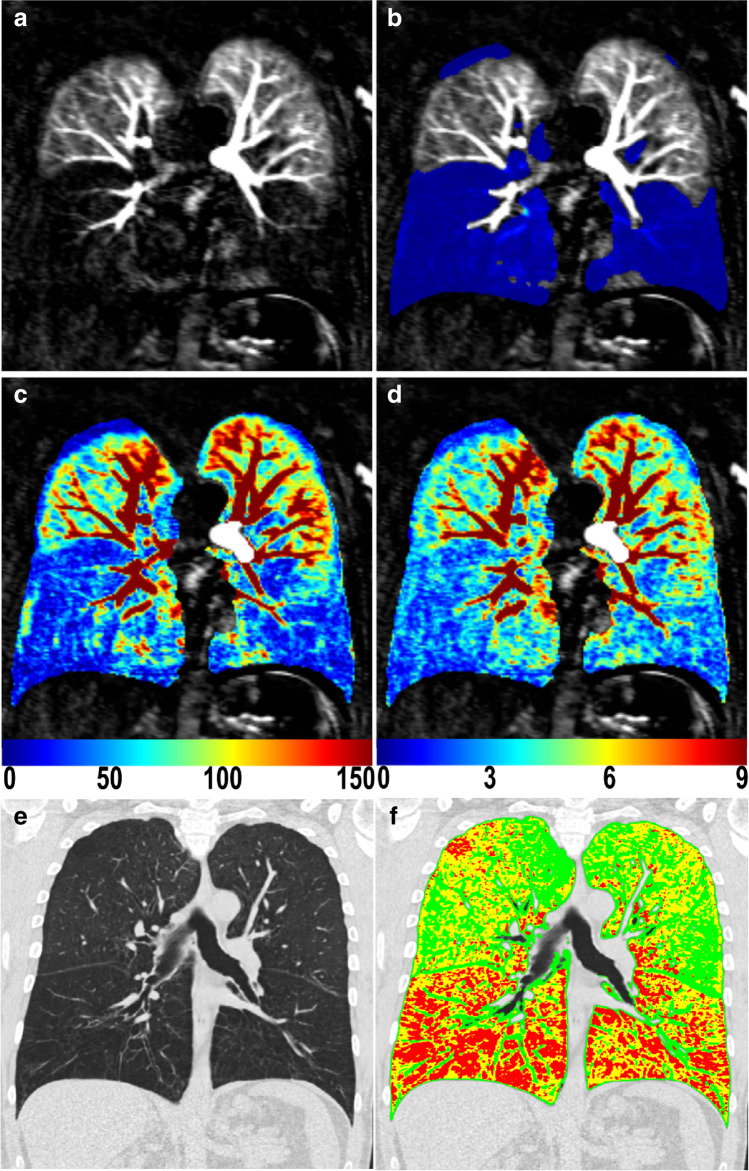
Representative DCE-MRI and CT of a 51 years old female patient with COPD GOLD2 with FEV1%predicted = 53.26%, FEV1/FVC = 0.53, and MRI perfusion score = 10. **a** Residue function map at the time point of maximum contrast-enhancement (*R*_max_ map), (**b**) corresponding map of perfusion defects in percent (QDP map, blue) calculated with Otsu’s method (QDP = 72.94%), (**c**) corresponding map of the pulmonary blood flow (PBF = 30.19 ml/100 ml/min), (**d**) corresponding map of the pulmonary blood volume (PBV = 2.34 ml/100 ml), (**e**) coronal CT and (**f**) corresponding parametric response map (PRM map) are presented. PRM classifies the voxels of the lung into normal lung tissue (28.23%, green), functional small airway disease (fSAD = 34.96%, yellow), and emphysema (36.13%, red)

The final cohort compromised 83 out of 103 subjects with evaluable baseline DCE-MRI (Table 1). Subjects were excluded before processing the MRI data, with 6 subjects due to missing MRI sequences, 11 subjects due to substantial respiratory artefacts, and 3 subjects due to failed CA application or other substantial artefacts. In 41 subjects, the DCE-MRI series were refined by manually removing acquisitions of time points with respiratory artefacts before or after the CA bolus passage through the lung, whereby on average 2.6 time points were removed. Lung segmentation masks were generated automatically in all cases. Representative MRI and CT images from a COPD patient with corresponding color-coded QDP, PBF, PBV, and PRM maps are given in Fig. 2.

**Table 1:** Patient demographics and baseline pulmonary function

	Total	At risk for COPD	Former GOLD 0	GOLD 1	GOLD 2	GOLD 3	GOLD 4	ANOVA *p* value
Demographics								
* n*	83	5	11	4	29	24	10	
Age (y)	65.7 ± 9.0	65.6 ± 6.8	69.5 ± 7.2	62.8 ± 9.5	66.4 ± 9.5	64.7 ± 10.2	62.7 ± 6.6	
Sex	44 f / 39 m	2 f / 3 m	7 f / 4 m	1 f / 3 m	15 f / 14 m	14 f / 10 m	5 f / 5 m	
Pack years	36.9 ± 28.8	32.2 ± 19.2	12.8 ± 13.0	23.0 ± 4.3	34.2 ± 24.2	39.6 ± 33.9	74.4 ± 22.5	
BMI (kg/m^2^)	26.2 ± 4.6	27.2 ± 6.5	29.5 ± 4.2	27.0 ± 4.9	25.8 ± 4.5	25.6 ± 4.7	23.9 ± 3.1	
Pulmonary function								
FEV1%predicted	55.9 ± 19.4	81.5 ± 10.2	78.2 ± 9.8	85.2 ± 2.4	61.1 ± 8.4	41.3 ± 5.2	26.3 ± 3.8	< 0.001
FEV1/FVC	0.56 ± 0.13	0.74 ± 0.03	0.76 ± 0.04	0.64 ± 0.03	0.58 ± 0.06	0.47 ± 0.08	0.37 ± 0.04	< 0.001

Information about pack-years was available for 45 patients only, BMI data were available for 80 patients only. Data are presented as mean ± standard deviation. *BMI* body mass index, *COPD* chronic obstructive pulmonary disease, *FEV1%predicted* forced expiratory volume in 1 s percent predicted, *FEV1/FVC* ratio between forced expiratory volume in 1 s and forced vital capacity

### Comparison between quantitative perfusion abnormalities and MRI perfusion score

**Fig. 3:**
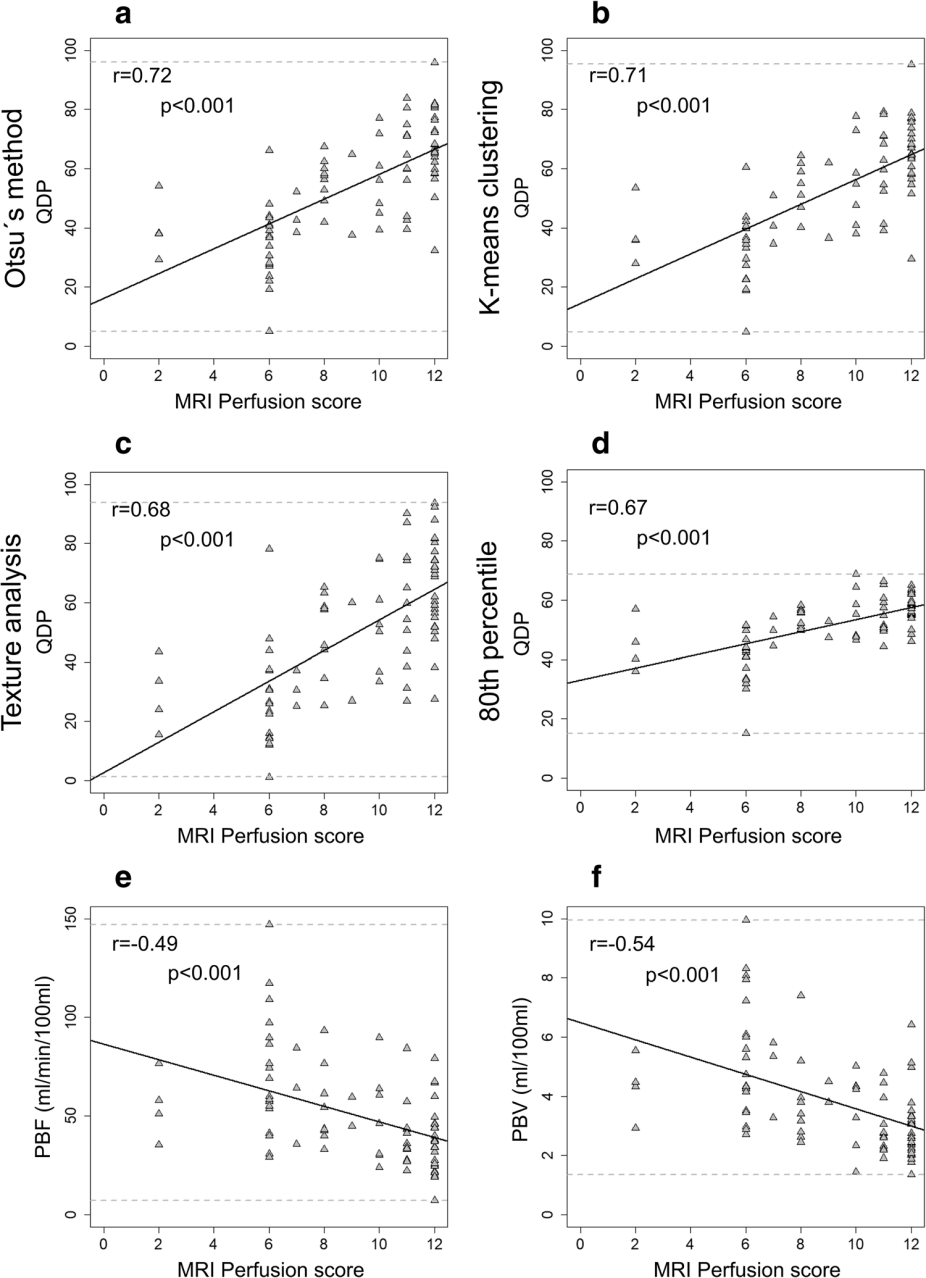
Association between perfusion defects in percent (QDP), pulmonary blood flow (PBF), and pulmonary blood volume (PBV) with MRI perfusion score. QDP calculated (**a**) based on Otsu’s method showed a range of values between 5.09 and 95.89%, (**b**) based on k-means clustering between 4.91 and 95.23%, (**c**) based on texture analysis between 1.28 and 93.73%, and (**d**) based on the 80^th^ percentile threshold between 15.17 and 68.82%, the latter being compression of the observed value range compared to the other QDP quantification methods. **e** PBF showed a range of observed values between 7.30 and 147.03 ml/min/100 ml and (**f**) PBV between 1.37 and 9.96 ml/100 ml. Respective linear regression lines, Spearman correlation coefficients, and corresponding *p* values are given in the plots

Mean values for the MRI perfusion parameters per subject group are given in Table 2. All MRI perfusion parameters increased (MRI perfusion score, QDP) or decreased (PBF and PBV) as expected with increasing GOLD severity (GOLD 1–4) (Table 2). The value range of QDP for the whole lung using Otsu’s method, k-means clustering, and texture analysis covered approximately 90% of the maximum theoretical range from 0 to 100%, whereas a smaller value range coverage was observed for the 80^th^ percentile threshold of approximately 53%. The correlation of QDP with the MRI perfusion score for the whole lung was moderate to strong (*r* = 0.68 to 0.72, *p* < 0.001) and the correlations of PBF and PBV with the MRI perfusion score were moderate (*r* = − 0.49 and *r* = − 0.54, *p* < 0.001). (Table 3, Fig. 3). Of note, QDP correlated significantly higher (Pearson and Filon’s z) with the MRI perfusion score than PBF and PBV (*p* < 0.001–0.05).

**Table 2:** Imaging results per subject group

	Total	At risk for COPD	Former GOLD 0	GOLD 1	GOLD 2	GOLD 3	GOLD 4	ANOVA *p* value
MRI visual scoring								
MRI perfusion score	9.1 ± 2.9	8.4 ± 2.3	7.3 ± 3.0	7.2 ± 2.5	9.2 ± 3.1	9.5 ± 2.7	11.2 ± 1.7	< 0.01
Quantitative DCE-MRI parameters								
QDP - Otsu’s method	54.6 ± 17.8	42.0 ± 15.0	39.0 ± 10.3	32.4 ± 23.5	57.3 ± 16.2	60.4 ± 14.4	65.5 ± 16.8	< 0.001
QDP - k-means clustering	52.7 ± 17.7	40.0 ± 14.7	36.7 ± 11.4	30.7 ± 21.7	55.2 ± 15.6	58.5 ± 14.2	64.6 ± 17.2	< 0.001
QDP - texture analysis	49.9 ± 22.6	36.8 ± 18.2	30.6 ± 17.8	24.7 ± 21.1	54.5 ± 22.2	55.1 ± 19.0	61.9 ± 19.9	< 0.001
QDP - 80^th^ percentile	51.7 ± 9.3	44.9 ± 9.1	43.7 ± 7.4	38.9 ± 17.2	52.7 ± 7.6	55.3 ± 7.1	57.7 ± 5.3	< 0.001
PBF (ml/100 ml/min)	50.6 ± 24.8	63.9 ± 27.1	65.9 ± 26.1	66.9 ± 23.4	44.5 ± 21.3	48.5 ± 27.0	42.8 ± 18.9	< 0.01
PBV (ml/100 ml)	3.8 ± 1.7	4.6 ± 1.7	5.2 ± 1.7	5.5 ± 3.2	3.5 ± 1.2	3.5 ± 1.7	3.0 ± 1.3	< 0.001
CT parametric response mapping								
PRM_Normal_ (%)	51.4 ± 21.4	80.1 ± 9.6	77.1 ± 21.4	63.2 ± 12.5	51.2 ± 17.0	40.2 ± 11.0	27.0 ± 7.3	< 0.001
PRM_Emph_ (%)	13.7 ± 11.7	5.0 ± 4.6	5.1 ± 8.4	5.9 ± 6.4	12.2 ± 10.3	16.4 ± 10.1	31.0 ± 7.8	< 0.001
PRM_fSAD_ (%)	34.4 ± 13.7	14.6 ± 5.6	17.3 ± 13.5	30.1 ± 6.2	35.8 ± 10.8	42.9 ± 9.1	41.6 ± 6.5	< 0.001
PRM_Abnormal_ (%)	48.6 ± 21.4	19.9 ± 9.6	22.9 ± 21.4	36.8 ± 12.5	48.8 ± 17.0	59.8 ± 11.0	73.0 ± 7.3	< 0.001

CT parametric response mapping data were only available for 76 patients, Data are presented as mean ± standard deviation. *COPD* chronic obstructive pulmonary disease, *Emph* emphysema, *fSAD* functional small airways disease, *PBF* pulmonary blood flow, *PBV* pulmonary blood volume, *QDP* perfusion defects in percent, *PRM* parametric response mapping

**Table 3:** Comparison of quantitative DCE-MRI perfusion parameters with the MRI perfusion score

	QDP				PBF (ml/100 ml/min)	PBV (ml/100 ml)
	Otsu´s method	K-means clustering	Texture analysis	80^th^ percentile		
MRI perfusion score whole lung						
* r*	0.72***	0.71***	0.68***	0.67***	− 0.49***	− 0.54***
|95% CI|	0.54, 0.78	0.55, 0.78	0.52, 0.77	0.48, 0.75	0.27, 0.61	0.31, 0.64
Mean diff	− 2.31 ± 2.13	− 2.50 ± 2.13	− 2.76 ± 2.38	− 0.13 ± 2.25	− 5.02 ± 4.22	− 4.52 ± 4.28
MRI perfusion score lobe-based						
Cohen´s kappa (k)	0.48	0.47	0.46	0.39		
%Agreement (%)	73.29	72.69	70.28	68.67		
Wilcoxon signed-rank test	0.06	0.27	< 0.05	0.38		

For the mean difference analysis in the whole lung, the quantitative values (QDP, PBF, PBV) were normalized to a maximum of 12 for statistical reasons. For comparison by lobe, QDP were converted into discrete values of 0, 1, and 2 at lobe level analogous to the visual scoring-system (Supplement Table E2) [30]. PBF and PBV could not be transferred to discrete values per lobe. *p* values smaller than 0.05 in the Wilcoxon signed rank test to evaluate the symmetry of the differences are considered to indicate a non-symmetrical distribution of the differences. *95% CI* 95% of confidence intervals, *Mean diff* mean difference ± standard deviation, *PBF* pulmonary blood flow, *PBV* pulmonary blood volume, *QDP* perfusion defects in percent, *%Agreement* percent agreement. *p < 0.05, **p < 0.01, and ***p < 0.001

In a lobe-based comparison between QDP, using converted discrete values, and MRI perfusion score Cohen’s k revealed moderate agreements, ranging between 0.39 for the 80^th^ percentile threshold and 0.48 for Otsu’s method. A bias in the symmetrical distribution in the differences between QDP and MRI perfusion score was evaluated with the Wilcoxon signed-rank test. A bias was observed for QDP quantified with texture analysis (Table 3).

### Comparison between MRI perfusion and CT parametric response mapping

CT PRM abnormalities increased with increasing GOLD severity (GOLD 1–4) (Table 2). All QDP quantification methods correlated strongly with PRM_Emph_ (*r* = 0.75 to 0.70, *p *< 0.001) and moderately with PRM_Abnormal_ (*r* = 0.65 to 0.61, *p* < 0.001), but only weakly with PRM_fSAD_ (*r *= 0.34 to 0.37, *p* < 0.01). In comparison, PBF and PBV were moderately correlated with PRM_Emph_ (*r* = − 0.51 and *r* = − 0.64, *p* < 0.001, respectively) and PRM_Abnormal_ (*r *= − 0.51 and *r* = − 0.63, *p* < 0.001, respectively), but weakly with PRM_fSAD_ (*r* = − 0.38 and *r* = − 0.40, *p *< 0.001, respectively) (Table 4). Of note, QDP based on Otsu’s method and k-means clustering correlated significantly higher (Pearson and Filon’s *z*) with PRM_Emph_ and PRM_Abnormal_ than the MRI perfusion score, PBF, and PBV.

**Table 4:** Comparison of DCE-MRI perfusion parameters with CT parametric response mapping and pulmonary function parameters

	MRI perfusion score	QDP				PBF (ml/100 ml/min)	PBV (ml/100 ml)
		Otsu’s method	K-means clustering	Texture analysis	80th percentile		
PRM_Abnormal_ (%)							
* r*	0.50***	0.63***	0.65***	0.62***	0.61***	− 0.51***	− 0.63***
|95 % CI|	0.30, 0.65	0.48, 0.76	0.49, 0.78	0.46, 0.75	0.42, 0.74	0.32, 0.66	0.48, 0.75
Mean diff	-	6.03 ± 16.94	4.15 ± 16.39	1.47 ± 19.30	3.23 ± 17.08	-	-
PRM_Emph_ (%)							
* R*	0.56***	0.74***	0.75***	0.70***	0.70***	− 0.51***	− 0.64***
|95% CI|	0.38, 0.69	0.62, 0.83	0.62, 0.84	0.55, 0.81	0.56, 0.80	0.32, 0.66	0.50, 0.77
Mean diff	-	40.4 ± 13.28	38.53 ± 12.91	35.85 ± 17.85	37.61 ± 9.17	-	-
PRM_fSAD_ (%)							
* r*	0.29**	0.35**	0.37**	0.37**	0.34**	− 0.38***	− 0.40***
|95% CI|	0.07, 0.46	0.12, 0.55	0.15, 0.55	0.14, 0.55	0.11, 0.55	0.16, 0.55	0.19, 0.58
Mean diff	-	19.68 ± 17.67	17.81 ± 17.28	15.12 ± 21.56	16.88 ± 12.79	-	-
FEV1%predicted							
* r*	− 0.39***	− 0.43***	− 0.44***	− 0.37***	− 0.49***	0.26*	0.38***
|95% CI|	0.18, 0.56	0.22, 0.59	0.25, 0.61	0.15, 0.54	0.32, 0.64	0.04, 0.45	0.19, 0.54
FEV1/FVC							
* r*	− 0.45***	− 0.50***	− 0.51***	− 0.41***	− 0.54***	0.28**	0.40***
|95% CI|	0.26, 0.62	0.31, 0.65	0.32, 0.65	0.21, 0.58	0.37, 0.68	0.07, 0.47	0.20, 0.57

Only for 76 patients CT parametric response mapping data were available. *Emph* emphysema, *FEV1%predicted* forced expiratory volume in 1 s percent predicted, *FEV1/FVC* ratio between forced expiratory volume in 1 s and forced vital capacity, *fSAD* functional small airways disease, *Mean diff* mean difference ± standard deviation, *PBF* pulmonary blood flow, *PBV* pulmonary blood volume, *QDP* perfusion defects in percent, *PFT* pulmonary function testing, *PRM* parametric response mapping. *p < 0.05, **p < 0.01, and ***p < 0.001

**Fig. 4:**
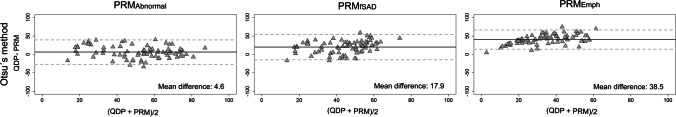
Bland–Altman plot between perfusion defects in percent (QDP) based on Otsu’s method using DCE-MRI and CT parametric response mapping (PRM) indices. Solid lines represent mean differences and dashed lines represent limits of agreements (+ -1.96SD) between QDP calculated with Otsu’s method and the PRM indices abnormal lung (PRM_Abnormal_), functional small airways disease (PRM_fSAD_), and emphysema (PRM_Emph_). Please note that the mean difference between QDP and PRM_Abnormal_ is close to zero. For the other three QDP calculation approaches, i.e., k-means clustering, texture analysis, and 80^th^ percentile threshold, the Bland–Altman plots are depicted in Supplementary Fig. 2

In Bland–Altman analyses, QDP was distinctly higher than PRM_Emphysema_ (mean difference QDP-PRM_Emph_ = 35.85 ± 17.85 to 40.4 ± 13.28) and PRM_fSAD_ (mean difference QDP-PRM_fSAD_ $=$ 15.12 ± 21.56 to 19.68 ± 17.67). When considering emphysema and fSAD together (PRM_Abnormal_), the mean difference was reduced and close to zero (mean difference QDP-PRM_Abnormal_ = 1.47 ± 19.30 to 6.03 ± 16.94) (Table 4, Fig. 4, and Supplementary Fig. 2).

### Comparison between MRI perfusion and pulmonary function testing

QDP and the MRI perfusion score correlated moderately with FEV1/FVC (*r* = − 0.41 to − 0.54, *p* < 0.001). Only a weak correlation of *r* = 0.28 (*p* < 0.05) and a moderate correlation of *r* = 0.40 (*p* < 0.001) were found for PBF and PBV, respectively (Table 4).

No statistically significant differences were observed between the correlation coefficients of QDP and the MRI perfusion score with PFT. However, QDP correlated significantly higher with FEV1/FVC than PBF (except for QDP based on 80^th^ percentile). QDP based on k-means clustering correlated significantly higher with FEV1/FVC than PBV.

## Discussion

We developed new methods to quantify QDP using DCE-MRI, which combine the advantages of unsupervised image clustering algorithms and mathematical models of tracer kinetics. Our results showed that (a) QDP based on Otsu’s method showed the highest consistency with the MRI perfusion score and (b) QDP is clinically meaningful due to its significant associations with the MRI perfusion score, CT PRM indices of emphysema and fSAD, and PFT. Furthermore, assessing pulmonary perfusion abnormalities as “defect-percent” showed advantages over the assessment of PBF and PBV, as we observed higher correlations with CT PRM indices and PFT for QDP.

The quantification of the conventional perfusion metrics PBF and PBV is based on R(t) map of the lung, which is often subject to high variability due to limited temporal resolution [35], non-linearity of the CA concentration to signal relationship [6, 7], overall low contrast-to-noise-ratio in the lung parenchyma, and respiratory motion artefacts. Consequently, this leads to physiologically undesirable and unreliable values in the R(t) map, which, in turn, increases the variability of PBF and PBV [8]. QDP uses the same R(t)map as a basis for the calculation and thus exploits the advantages of mathematical models based on the principles of tracer kinetics for non-diffusible tracers. However, QDP does not use the individual voxel values such as PBF and PBV as the next step, but instead uses unsupervised image clustering algorithms to identify poorly-perfused voxels. Hence, the influence of certain challenges of DCE-MRI sequences in the lungs is reduced, and thus, the calculation should be more robust. Overall, we observed higher correlations of QDP with the MRI perfusion score, CT PRM indices, and PFT compared to PBF and PBV.

Since QDP is determined using an automated computer algorithm, the calculation is time-efficient and allows for a more detailed assessment of perfusion abnormalities compared to visual MRI perfusion scoring. Furthermore, it is user-independent and eliminates potential intra- and inter-reader variabilities [2, 19]. In this study, QDP was comparable to the MRI perfusion score and showed even higher correlations with CT PRM indices and PFT.

We compared four different QDP quantification approaches to assess the strengths and weaknesses of each approach. We not only mainly developed and optimized QDP to reflect the MRI perfusion score, but also compared the different QDP methods for method development purposes with CT PRM and PFT parameters, since perfusion abnormalities are associated with both airflow limitations and the destruction of lung parenchyma. We propose to use QDP based on Otsu’s method for future clinical studies, because it showed overall the highest level of agreement with the MRI perfusion score and high correlations with CT PRM and PFT parameters. However, it must be mentioned that all presented clustering approaches require a predefined number of classes, which implies that the methods must find perfusion defects and well-perfused tissue. Consequently, clustering approaches may potentially overestimate mild disease and underestimate very severe disease. Minor perfusion defects under 7.5% should be rejected as no perfusion defects, similar to what was done in the lobe-based comparison [30]. In previous studies, percentile thresholds were used to quantify pulmonary perfusion defects using DCE-MRI [12, 36]. We noticed a compression of the range of observed values for the percentile method compared to the other methods, which is caused by its underlying calculation method, regardless of the used percentile and factor. This compressed value range affects the comparability with the MRI perfusion score. The percentile and factor used here were empirically determined in a previous study [36] and may not be transferable to data of other MRI acquisition techniques or scanner types. The use of texture analysis or other more advanced analysis methods might be challenging due to the low contrast-to-noise ratio and pronounced artefacts in DCE-MRI of the lungs.

The general concept of describing functional lung abnormalities as “defect-percent” appears to be clinically meaningful as ventilation defects in percent quantified from hyperpolarized gas MRI have already been used to monitor treatment response in CF [37, 38]. Initial studies on the quantification of pulmonary perfusion as “defect-percent” using DCE-MRI and FD-MRI also indicate its potential, but further evaluation was missing to date [12, 30]. The quantification of perfusion defects may provide complementary or overlapping information to ventilation defects, because pulmonary perfusion and ventilation are related through the HPV. However, studies are indicating that the ventilation-perfusion match in COPD deteriorates with an increasing level of emphysema [39]. Here the ability to block the HPV in inflamed lung regions seems to be suspended in emphysema-susceptible patients and may therefore contribute to emphysema development [40]. In addition, studies in COPD could already demonstrate that fSAD precedes the development of emphysema using CT [41, 42]. Consequently, the comparison of perfusion abnormalities with PRM_fSAD_ can confirm the hypothesis that perfusion abnormalities may serve as a prognostic biomarker for emphysema progression in COPD. Moreover, it was speculated whether impaired pulmonary perfusion is a reversible component of the COPD pathogenesis [1], which was already demonstrated in patients with CF [20] and COPD [43]. In a previous study, no association between PBF using DCE-MRI and small airways disease using CT was observed [1]. We observed significant correlations between QDP and the PRM parameters PRM_Emph_, PRM_fSAD_, and PRM_Abnormal_. In addition, the extent of perfusions defects from MRI corresponds to the CT-derived extent of abnormal lung (emphysema and fSAD). Considered separately, more perfusion defects were observed than emphysema or fSAD. This indicates that QDP represents the entire parenchymal abnormalities detected by PRM.

We found moderate correlations between QDP and FEV1%predicted or FEV1/FVC, indicating a relationship between perfusion abnormalities and airflow limitation. This is consistent with previous studies [12, 44] and in agreement with the understanding that a reduction in FEV1%predicted is mainly driven by large airway obstruction, whereas pulmonary perfusion abnormalities probably reflect also small airway pathologies [1, 20].

One technical limitation of this study is the use of data from one single scanner. Other QDP quantification methods, or PBF and PBV, could be superior in future studies with different patient populations and/or other DCE-MRI sequences with better image quality. The DCE-MRI quality issues in the lung are primarily caused by the measurement technique to achieve the necessary temporal resolution, together with the low proton density and pronounced susceptibility artefacts in the lungs. These issues even get worse with disease severity in COPD. Furthermore, the long acquisition time in inspiratory breath-hold is challenging for many COPD patients. The acquisition time is pre-defined as individual circulation times of the CA through the lungs are unknown. However, only the time points of the first CA-bolus passage through the lungs are required for the perfusion quantification. The removal of time points affected by respiratory motion could influence the PBF and PBV quantification (absolute flow and volume values), but distinctly less the QDP calculation (relative values due to the intrinsic normalization of the clustering algorithms). Attempts to correct respiratory motion with image registration have not succeeded so far. Since cross-sectional correlations do not prove causality, the relevance of perfusion abnormalities for the development of emphysema must be further investigated in large longitudinal studies, ideally with a regional comparison between quantitative CT and quantitative MRI. In addition, interventional studies are prerequisites before QDP can be used in clinical drug development and the robustness and repeatability of the quantitative perfusion parameters must be assessed in further method validation studies.

This study demonstrates QDP quantified using DCE-MRI is associated with established markers of disease severity in COPD. It corresponds to the extent of emphysema and fSAD in percent quantified using CT, indicating that pulmonary perfusion abnormalities themselves may contribute to or at least precede the development of irreversible emphysema. QDP showed considerable advantages over PBF and PBV, which were used in previous COPD studies. We conclude that QDP based on Otsu’s method from DCE-MRI is a promising novel biomarker for clinical trials in COPD.

## Supplementary Information

Below is the link to the electronic supplementary material.

Supplementary file1 (DOCX 898 KB)

## Abbreviations

AIF: Arterial input function
ANOVA: One-way analysis of variance
CA: Contrast agent
CCA: Cross-correlation analysis
COPD: Chronic obstructive pulmonary disease
DCE-MRI: Dynamic contrast-enhanced magnetic resonance imaging
FD: Fourier decomposition
FEV1%predicted: Forced expiratory volume in 1 s percent predicted
FEV1/FVC: Ratio between forced expiratory volume in 1 s and forced vital capacity
fSAD: Functional small airways disease
HPV: Hypoxic pulmonary vasoconstriction
PBF: Pulmonary blood flow
PBV: Pulmonary blood volume
PFT: Pulmonary function testing
PRM: Parametric response mapping
QDP: Perfusion defects in percent
Rmax map: Residue function map at the time point of maximum contrast enhancement
R(t) map: Time-resolved residue function map

## Declarations

### Guarantor

The scientific guarantor of this publication is Prof. Dr. med. Mark O. Wielpütz, EDIR, MHBA.

### Statistics and biometry

Carina Ittrich kindly provided statistical advice for this manuscript.

### Methodology

• retrospective

• cross-sectional study

• performed at one institution
