# Supplementary material for: Quantification of pulmonary perfusion abnormalities using DCE-MRI in COPD: comparison with quantitative CT and pulmonary function
Source: Eur Radiol. 2021 Sep 22;32(3):1879–90. doi: 10.1007/s00330-021-08229-6 (PMC8831348; doi:10.1007/s00330-021-08229-6)
Supplement: Supplementary file 1 — Supplementary file1 (DOCX 898 KB) [file 330_2021_8229_MOESM1_ESM.docx]

**Supplementary materials**

**MRI quantification pipeline**

*Exclusion of voxels affected by respiratory artefacts*

The identification of voxels affected by respiratory motion was performed via normalized cross-correlation analysis (CCA). The time-resolved signal of such voxels in the subtraction image has a different shape than the AIF, or the signal maximum occurs with a high time-shift compared to the signal maximum of the AIF. The method is based on the previously published principles for suppressing the pulmonary vasculature from DCE-MRI [1]. The normalized CCA was calculated for the whole time period to evaluate curve similarity and time-shift together, as well as for each time step to evaluate curve similarity and time-shift separately. Voxels with low correlation coefficients and a late correlation maximum, were considered as corrupted by respiratory motion and thus excluded. Additionally, voxels with unreliable high values in PBV over 200ml/100ml in the lungs were excluded. After the exclusion process, holes in the lung segmentation mask were closed automatically. As a result, only voxels affected by respiratory motion at the border of the segmentation mask were removed, which includes the diaphragm area.

*Automatic lung segmentation*

The lungs were segmented from coronal and transversal T1-weighted gradient-echo volume-interpolated breath-hold examination (VIBE, Siemens Healthineers) images because of the significantly higher spatial resolution and contrast between the lung and the chest wall compared to the DCE-MRI. The entire chest was separated from the background, and seed points were identified in each lung using histogram analysis. The lung segmentation mask was generated by region-growing, from which the trachea was excluded, as previously described [2]. As a modification to the approach published by Kohlmann et al., a watershed transformation was used to ensure a clear separation of right and left lung, the histogram thresholds were adapted, and only the trachea until the bifurcation was excluded from the lung segmentation mask. The lung segmentation mask was registered with a multimodality non-rigid demon algorithm [3] to the first time point of the DCE-MRI data before CA arrival to avoid interference through contrast enhancement. A modality transformation [4] was performed before the pixel-based non-rigid demon registration to compensate for the different tissue contrasts between the MRI pulse sequences [5–7].

*Quantification of pulmonary perfusion abnormalities*

All perfusion quantification approaches were performed using the three-dimensional R_max_ map in total and not individual slices separately. The time point of the maximum contrast enhancement in the R(t) map was determined by averaging the R(t) map arithmetically over the whole lung at each time point, except the first time point to avoid artefacts. The R_max_ maps were median filtered to reduce noise and remove isolated voxels while preserving edges. All voxels outside of the lung were set to zero.

Otsu´s method and k-means clustering

Otsu's method using intensity histograms [8] and k-means clustering were utilized to separate the voxels in the lung mask of the R_max_ map in poorly-perfused lung voxels, well-perfused lung voxels, and vessels, whereby the poorly-perfused lung voxels were classified as perfusion abnormalities. As Otsu´s method is sensitive to variations in the ratio between object and background [9, 10], the background size was adjusted to the size of the object, hence the lung mask. The k-means clustering was performed using the complete R_max_ map, including the zeroed background.

The underlying calculation principle is equivalent for k-means clustering and Otsu’s method, i.e., minimize the intra-class variance. The differences between the two methods are particularly noticeable in the required image pre-processing effort.

Texture analysis

For the image classification based on texture features, a grey-level co-occurrence matrix [11] with 32 classes, an offset of [0 1 0] and symmetry condition across the diagonal of the co-occurrence matrix was applied to the whole R_max_ map. The class with the lowest mean intensity from the R_max_ map was classified as perfusion abnormalities, all other levels as well-perfused or vessels.

80^th^ percentile threshold

The percentile threshold approach divided the voxels in the R_max_ map into well-perfused voxels and poorly-perfused voxels. The threshold between the two classes was calculated by multiplying the 80th percentile of the R_max_ map intensity within the lung mask with 0.5 as proposed by Bauman et al. [12].

The presented clustering approaches, number of classes and the use of the R_max_ map were determined through an optimization process. For this purpose, QDP was calculated using several other numbers of classes, clusters, textures and other percentile thresholds multiplied with several factors. In addition to the R_max_ map, QDP was also calculated from subtraction images at maximum, PBF map, and PBV map during the optimization process. The presented specifications for the respective QDP methods were chosen because they showed the highest agreement with the MRI perfusion score.

In this study no correction of the perfusion gradient from anterior to posterior, which is caused by the patient’s supine position in the MR scanner, was implemented, to increase the comparability of the four QDP methods [12–14].

**Statistical analysis**

The Spearman correlation coefficient was rated as suggested by Karlik for radiological features: 0.0-0.2 as very weak, 0.2-0.4 as weak, 0.4-0.7 as moderate, 0.7-0.9 as strong, and 0.9-1.0 as very strong [15]. Cohen's k was calculated with the condition that all levels of disagreement between raters are weighted equally. Correlation coefficients were compared statistically with the R-package “cocur” [16], whereby the p-values from Pearson and Filon's z are given [17]. The correlation coefficient comparison was performed using the one-tailed test, to identify if the correlation coefficient is significant greater.


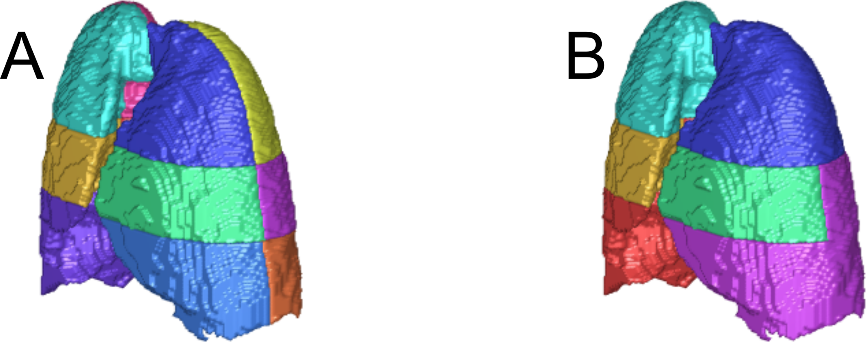


**Supplementary fig. 1.** Color-coded 3D visualization of the automatic lung partitioning process. A) First, the lung was divided into twelve regions of equal volume (six per lung) (20). B). Second, the six regions of equal size per lung were combined to approximate the three lobes per lung as follows: upper lobe=both upper regions, middle lobe or lingula=ventral middle region, and lower lobe=dorsal middle region and both lower regions.


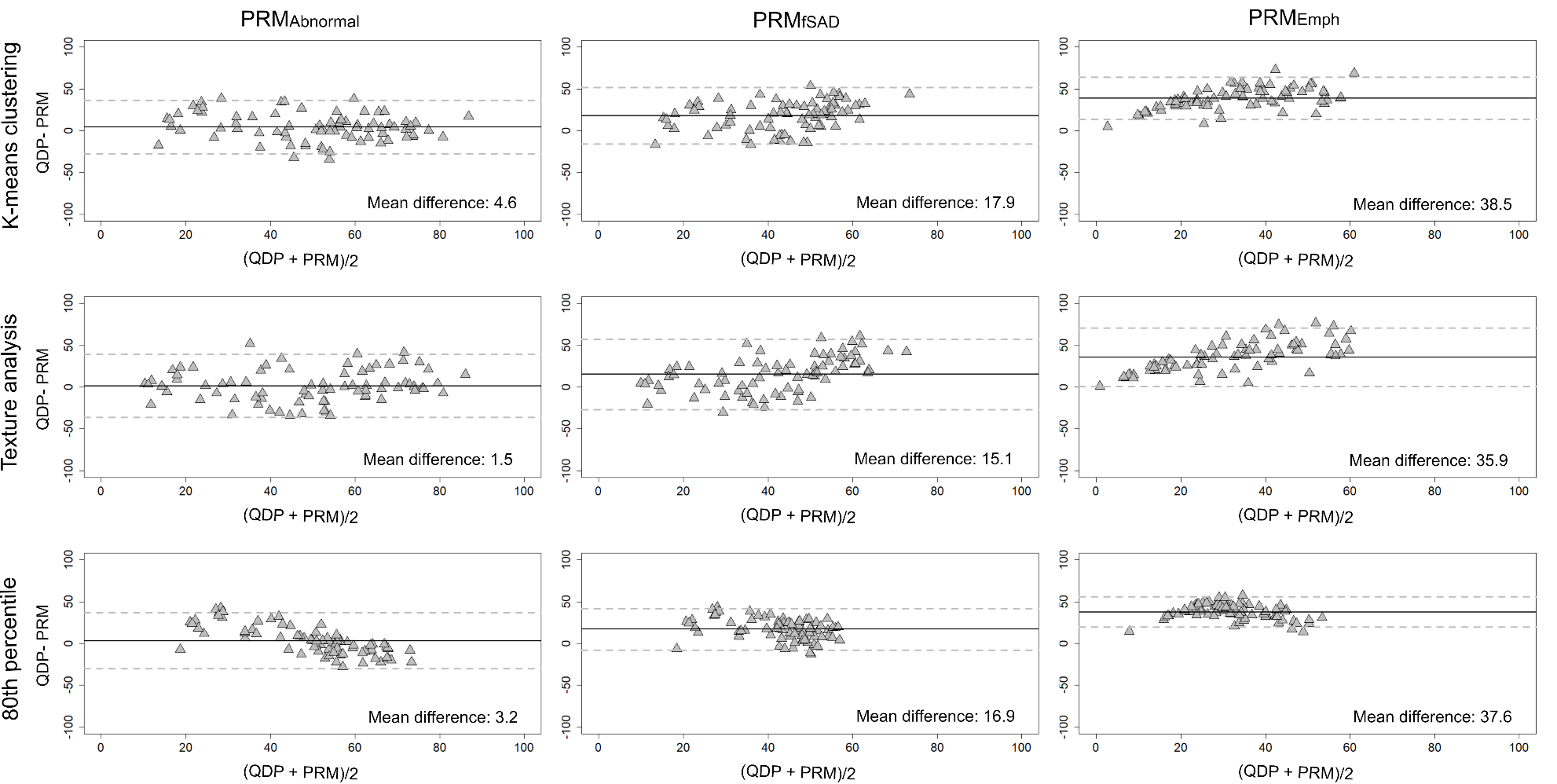


**Supplementary fig. 2.** Bland-Altman plots of the observed differences between perfusion defect percent (QDP) based on k-means clustering, texture analysis and 80^th^ percentile threshold using DCE-MRI and CT parametric response mapping (PRM). Solid lines represent mean differences and dashed lines represent limits of agreement (+-1.96SD) between QDP calculated with different approaches and the PRM indices abnormal lung (PRM_Abnormal_), functional small airways disease (PRM_fSAD_) and emphysema (PRM_Emph_). Please note that mean difference between QDP and PRM_Abnormal_ is close to zero.

**Supplementary table 1** Standardized MRI protocol [18]

| **MRI pulse sequence** | **Specials** | **Plane** | **TR (ms)** | **TE (ms)** | **ST (mm)** | **Distance factor (%)** | **Slices** | **FoV (mm^2^)** | **Matrix** | **Voxel size (mm)** | **Scan time (min:s)** |
| --- | --- | --- | --- | --- | --- | --- | --- | --- | --- | --- | --- |
| T1 GRE | bh | tra | 3.61 | 1.69 | 4.0 | 3D | 88 | 400x300 | 320x240 | 1.3 x 1.3 | 0:18 |
| T1 GRE | bh | cor | 3.35 | 1.63 | 4.0 | 3D | 56 | 400x400 | 288x288 | 1.4 x 1.4 | 0:16 |
| 4D Perfusion | 20 acquisitions, bh, Gd | cor | 1.80 | 0.74 | 5.0 | 3D | 44 | 450x366 | 256x208 | 1.8 x 1.8 | 0:38 |

T1 GRE = T1-weighted gradient echo (GRE) volume interpolated breath-hold examination (VIBE); 4D Perfusion = time-resolved 3D gradient echo sequence with parallel imaging and view sharing (time-resolved angiography with stochastic trajectories [TWIST]); Gd = i.v. injection of Gadolinium-based contrast material; tra = transversal plane; cor = coronal plane; bh = breath-hold; TR = repetition time; TE = echo time; ST = slice thickness; FoV = Field of view

**Supplementary table 2** Comparison of visual MRI perfusion score with automatically detected perfusion defects in percent

| **Discrete value** | **MRI perfusion score** | **QDP** |
| --- | --- | --- |
| **0** | Normal | <7.5 % |
| **1** | <50 % | ≥7.5 % to <50 % |
| **2** | ≥50 % | ≥50 % |

The discrete values 0, 1 or 2 were assigned per lobe to achieve comparability of the visual perfusion score with quantitative perfusion defects in percent (QDP). Accordingly, values from 0 to 12 can be achieved for the perfusion score and QDP for the whole lung. The threshold of <7.5% was defined for no perfusion defects in QDP to compensate errors caused by noise and image artifacts, as described previously [19].

**Supplemental references**

1. Risse F, Kuder TA, Kauczor H-U, et al (2009) Suppression of pulmonary vasculature in lung perfusion MRI using correlation analysis. Eur Radiol 19:2569. https://doi.org/10.1007/s00330-009-1464-9

2. Kohlmann P, Strehlow J, Jobst B, et al (2015) Automatic lung segmentation method for MRI-based lung perfusion studies of patients with chronic obstructive pulmonary disease. International journal of computer assisted radiology and surgery 10:403–17. https://doi.org/10.1007/s11548-014-1090-0

3. Kroon D-J (n.d.) Multimodality non-rigid demon algorithm image registration (https://www.mathworks.com/matlabcentral/fileexchange/21451-multimodality-non-rigid-demon-algorithm-image-registration). MATLAB Central File Exchange Retrieved July 10, 2018

4. Kroon D-J, Slump CH (2009) MRI MODALITIY TRANSFORMATION IN DEMON REGISTRATION. 2009 Ieee Int Symposium Biomed Imaging Nano Macro 963–966. https://doi.org/10.1109/isbi.2009.5193214

5. Thirion J-P (1998) Image matching as a diffusion process: an analogy with Maxwell’s demons. Med Image Anal 2:243–260. https://doi.org/10.1016/s1361-8415(98)80022-4

6. Cachier P, Pennec X, Ayache. N (1999) Fast Non Rigid Matching by Gradient Descent: Study and Improvements of the “‘Demons’” Algorithm. RR-3706, INRIA inria–00072962

7. Wang H, Dong L, O’Daniel J, et al (2005) Validation of an accelerated ‘demons’ algorithm for deformable image registration in radiation therapy. Phys Med Biol 50:2887–2905. https://doi.org/10.1088/0031-9155/50/12/011

8. Otsu N (1979) A Threshold Method from Gray-Level Histograms. Ieee Transactions Syst Man Cybern , vol 9, no 1 9:62–66. https://doi.org/10.1109/tsmc.1979.4310076

9. Lee SU, Chung SY, Park RH (1990) A comparative performance study of several global thresholding techniques for segmentation. Comput Vis Graph Image Process 52:171–190. https://doi.org/10.1016/0734-189x(90)90053-x

10. Kittler J, Illingworth J (1985) On threshold selection using clustering criteria. Ieee Transactions Syst Man Cybern SMC-15:652–655. https://doi.org/10.1109/tsmc.1985.6313443

11. Haralick RM, Shanmugam K, Dinstein I (1973) Textural Features for Image Classification. Ieee Transactions Syst Man Cybern SMC-3:610–621. https://doi.org/10.1109/tsmc.1973.4309314

12. Heimann T, Eichinger M, Bauman G, et al (2012) Automated scoring of regional lung perfusion in children from contrast enhanced 3D MRI. 83150U-83150U–6. https://doi.org/10.1117/12.911946

13. Hueper K, Parikh MA, Prince MR, et al (2013) Quantitative and semiquantitative measures of regional pulmonary microvascular perfusion by magnetic resonance imaging and their relationships to global lung perfusion and lung diffusing capacity: the multiethnic study of atherosclerosis chronic obstructive pulmonary disease study. Invest Radiol 48:223–30. https://doi.org/10.1097/rli.0b013e318281057d

14. Kaireit TF, Voskrebenzev A, Gutberlet M, et al (2019) Comparison of quantitative regional perfusion‐weighted phase resolved functional lung (PREFUL) MRI with dynamic gadolinium‐enhanced regional pulmonary perfusion MRI in COPD patients. J Magn Reson Imaging 49:1122–1132. https://doi.org/10.1002/jmri.26342

15. Karlik SJ (2003) Exploring and Summarizing Radiologic Data. Am J Roentgenol 180:47–54. https://doi.org/10.2214/ajr.180.1.1800047

16. Diedenhofen B, Musch J (2015) cocor: A Comprehensive Solution for the Statistical Comparison of Correlations. Plos One 10:e0121945. https://doi.org/10.1371/journal.pone.0121945

17. Pearson K, Filon LNG (1898) Mathematical contributions to the theory of evolution. IV. On the probable errors of frequency constants and on the influence of random selection on variation and correlation. P R Soc London 62:173–176. https://doi.org/10.1098/rspl.1897.0091

18. Triphan SMF, Biederer J, Burmester K, et al (2018) Design and application of an MR reference phantom for multicentre lung imaging trials. Plos One 13:e0199148. https://doi.org/10.1371/journal.pone.0199148

19. Bauman G, Puderbach M, Heimann T, et al (2013) Validation of Fourier decomposition MRI with dynamic contrast-enhanced MRI using visual and automated scoring of pulmonary perfusion in young cystic fibrosis patients. Eur J Radiol 82:2371–7. https://doi.org/10.1016/j.ejrad.2013.08.018
